# Solid-State [2+2] Photoreaction of Isostructural Cd(II) Metal Complexes and Solid-State Fluorescence

**DOI:** 10.3390/molecules29020351

**Published:** 2024-01-10

**Authors:** Akansha Ekka, Aditya Choudhury, Madhumita Samanta, Ayushi Deshmukh, Nathan R. Halcovitch, In-Hyeok Park, Raghavender Medishetty

**Affiliations:** 1 Department of Chemistry, Indian Institute of Technology Bhilai, Durg 491001, Indiaadityac@iitbhilai.ac.in (A.C.);; 2 Chemistry Department, Lancaster University, Lancaster LA1 4YB, UK; 3 Graduate School of Analytical Science and Technology (GRAST), Chungnam National University, Daejeon 34134, Republic of Korea

**Keywords:** [2+2] cycloaddition, isostructural complexes, Schmidt’s criteria, HH and HT alignments, 0D transformation

## Abstract

A green method to synthesize cyclobutane derivatives has been developed over the past three decades in the form of solid-state [2+2] photochemical reactions. These solid-state reactions also play a major role in the structural transformation of hybrid materials. In this regard, crystal engineering has played a major role in designing photoreactive molecular systems. Here, we report three novel binuclear Cd(II) complexes with the molecular formula [Cd_2_(4spy)_4_L_4_], where 4spy = 4-styryl pyridine and L = *p*-toluate (**1**); 4-fluorobenzoate (**2**); and 3-fluorobenzoate (**3**). Although three different benzoates are used, all three complexes are isostructural, as corroborated through SCXRD experiments. Structural analysis also helped in identifying two potential photoreactions. These are both intra- and intermolecular in nature and are driven by the head-to-head (HH) and head-to-tail (HT) alignment of 4spy linkers within these metal complexes. ^1^H NMR spectroscopy studies showed evidence of a quantitative head-to-head photoreaction in all these three complexes, and SCXRD analysis of the recrystallization of the photoproducts also provided confirmation. TGA studies of these photoreactive complexes showed an increase in the thermal stability of the complexes due to the solid-state photoreaction. Photoluminescence studies of these complexes have been conducted, showing a blue shift in emission spectra across all three cases after the photoreaction.

## 1. Introduction

Solid-state [2+2] photoreactions are of great interest due to their use of light energy to prepare strained organic molecules that are difficult to obtain through traditional routes, especially with quantitative yields [1,2,3,4,5,6,7,8]. Designing these solid-state reactions is traditionally done through non-covalent interactions and coordination bonds, among others [9,10,11,12,13,14]. In metal complexes, the coordination bond is important in aligning reactive centers and their reaction upon photoirradiation.

The criteria of solid-state [2+2] cycloaddition were postulated by Schmidt, wherein the photoreactive linker of neighboring groups is aligned in a ready-to-react manner to form a highly strained cyclobutane ring [15]. Most of these solid-state reactions are often used in structural transformations, where the specific reactions are executed through specific structural transformations to obtain *regio*- and *stereo*-specific products with quantitative yields. Obtaining these photoproducts in a similar *regio*- and *stereo*-specific manner with quantitative yields is challenging or almost impossible in regular solution-state synthesis [16,17,18,19,20]. It is very rare to observe the competition between the head-to-head and head-to-tail alignment of photoreactive linkers in a single complex. Observing these different alignments and studying these photoreactions is very difficult without the aid of single-crystal structural analysis. At the same time, single-crystal to single-crystal transformation is challenging because, during the structural change, the strain developed inside a single crystal causes it to pop violently so as to release the strain, thus resulting in the loss of its crystallinity [21,22,23]. The plausible photoreaction in these simple complexes is important mainly due to the better design and understanding of photoreactions, as well as the control over photo-transformation reactions [5,24,25]. 

Isostructural compounds and the study of their properties are highly significant, mainly due to the possibility of finetuning the materials’ properties without introducing significant structural changes. This is also one of the prerequisites for solid solutions, which are known for their capacity to finetune the properties of materials without significant modifications in *structure*–*property* relations. Metal ions with diverse geometries are valuable for constructing metal complexes and coordination polymers, offering properties such as optical characteristics, robustness and flexible frameworks [26,27]. Cadmium is particularly intriguing due to its ability to adopt various coordination modes and form complexes with diverse ligands. This flexibility stems from its capacity to exhibit different coordination numbers and predictable geometries, enabling the design of complexes with flexible structures and the finetuning of electronic properties and stability in coordination polymers [28]. Additionally, the diamagnetic nature of Cd(II) is advantageous for monitoring the photoreaction of olefin groups using NMR studies [29,30,31].

**Figure 1:**
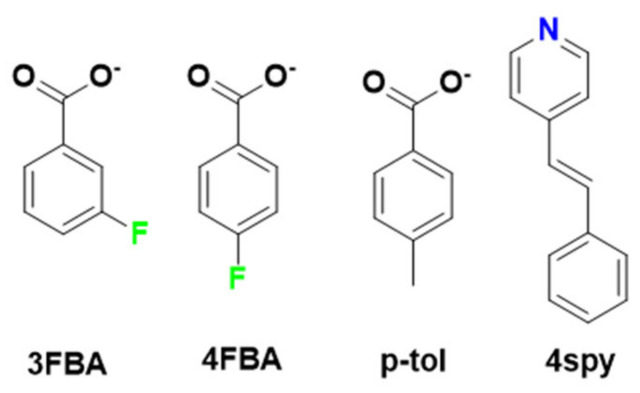
Structural diagrams of benzoate derivatives and linker 4spy.

In this article, we studied the photoreactivity of three binuclear Cd(II) metal complexes in the context of solid-state [2+2] photochemical reactions, using *p*-toluate(*p*-tol) and 4- and 3-fluorobenzoates as the carboxylate linkers, and 4spy as the photoreactive linker (Figure 1). All three complexes are isostructural in nature. The 4spy photoreactive linkers are aligned in both head-to-head and head-to-tail manner and follow Schmidt’s criteria. The photoirradiation of these compounds showed only head-to-head photoreactions between the 4spy linkers, which was confirmed both via the quantitative photoreaction and through single-crystal XRD analysis. Photoluminescence studies confirmed the blue shifting of the fluorescence peaks after the photoreaction. In addition, an increment in the thermal stability of the compounds was observed due to the photoreaction. 

## 2. Results and Discussions

### 2.1. Structural Description of [Cd_2_(p-tol)_4_(4spy)_4_] (***1***)

**Figure 2:**
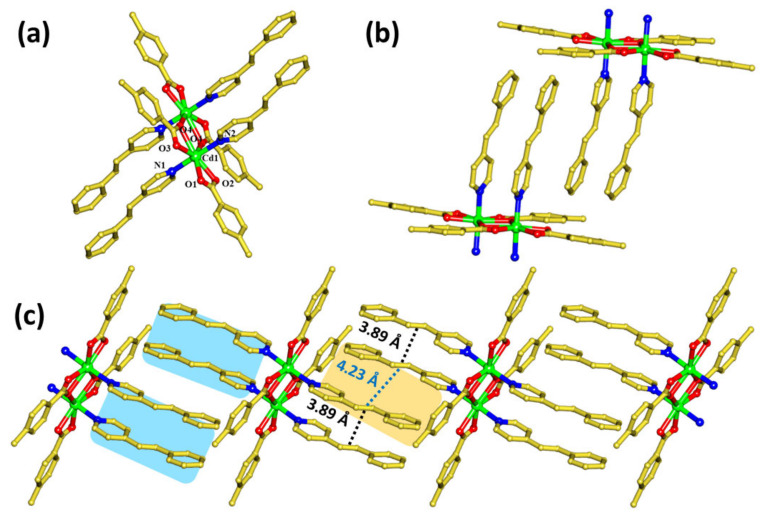
Crystal structure of [Cd_2_(*p*-tol)_4_(4spy)_4_] (**1**). (**a**) Metal complex of **1**. (**b**) Alignment of photoreactive linker in **1**. (**c**) Extended head-to-head and head-to-tail alignment. All H-atoms are omitted for clarity. Color codes: C = golden; N = dark blue; O = red; Cd = green.

Block-shaped yellow single crystals of **1** were obtained from the slow evaporation of a methanolic solution of Cd(ClO_4_)_2_·6H_2_O, *p*-tol and 4spy in 1:2:1 molar ratios, respectively, after a few days. The structure of this compound has been characterized through single-crystal XRD analysis. The results confirm that this compound is crystallized in the triclinic *P*-1 space group with *Z* = 1. The asymmetric unit constitutes exactly half the formula unit of the compound. The Cd(II) atoms are coordinated by five oxygen atoms from three *p*-tol linkers at equatorial positions, where four O-atoms from two *p*-tol linkers are chelated around one metal center, while the fifth O-atom bridges between two Cd(II) metal centers, as shown in (Figure 2a). The center of symmetry of this compound is present in the middle of the two Cd(II) atoms. The apical sites of this Cd(II) pentagonal planar geometry are bonded by two 4spy linkers through *N*-atoms. This bimetallic node, commonly observed with Cd(II) atoms, is one of the most prevalent and well-documented nodes [29,30,31]. The phase purity of the compound has been characterized through the comparison of PXRD data (see Figures S1–S3 in Supplementary Materials). 

Due to the bimetallic nature of the metal complex, with the distance between the Cd(II) atoms of 3.86 Å, the 4spy linkers are expected to be present in close proximity. As per expectation, the centers of olefinic units of the photoreactive linkers are arranged in a head-to-head (HH) manner and separated by a distance of 3.89 Å (Figure 2b,c). The olefinic hydrogen and the hydrogen of phenyl group interact with the carboxyl oxygen of *p*- tol (C-H···O = 2.42 Å and C-H···O = 2.51 Å, 2.49 Å respectively). Also, the phenyl group of *p*-tol interacts with the phenyl hydrogen of 4spy (C-H···π = 2.88 Å). The intramolecular π-π interactions between the pyridyl group and the phenyl group, with distances 3.77 Å and 3.86 Å, respectively, are observed (Figure S8).

Further analysis of the structure revealed that one of the two 4spy linkers that are attached to the Cd(II) atoms are aligned with the neighboring molecule’s 4spy linker in a head-to-tail (HT) manner, where the centers of the olefin groups of these 4spy linkers are separated by a distance of 4.23 Å (Figure 2c). Both the head-to-head and the head-to-tail alignments of the olefin groups are near or within Schmidt’s topochemical criteria [15]. These criteria have been mainly established through the observation of the photoreactivity of various cinnamic acid derivatives, as well as the separation of olefins and their photoreaction. Meanwhile, this criterion also suggests that there is a significant interaction among the *p_z_* orbitals of olefin carbons within 4.2 Å; hence, the observation of photoreaction is facilitated under UV illumination. Thus, the photoreaction of olefins in these complexes can occur through two possibilities, based on the alignment of 4spy linkers and their reactive olefin units. 

The first possibility is the photoreaction of 4spy linkers at the intramolecular level, and the other possibility is the photoreaction of the 4spy linkers between the neighboring molecules (Figure 2c). In the first case, the transformation is a 0D to 0D transformation, whereas the second transformation could lead to the photo-polymerization of metal complexes to a 1D coordination polymer (CP) (0D to 1D transformation). In the case of photo-polymerization of metal complexes, only one of the 4spy linkers that is attached to the Cd(II) is aligned and could undergo photoreaction; this photo-transformation could lead to only a 50% photoreaction, whereas another 4spy linker is expected to be inert (Figure 2c, blue shade). However, in the case of the intramolecular photoreaction, both linkers that are attached to Cd(II) would undergo photo-cycloaddition (Figure 2c, orange shade). This 0D-to-0D transformation results in a quantitative photoreaction. In other words, the percentage of the photo-cycloaddition reaction could help in the assessment of the type of photoreaction and reactive linkers in the metal complexes [32,33,34,35].

### 2.2. Photoreactivity of ***1***

**Figure 3:**
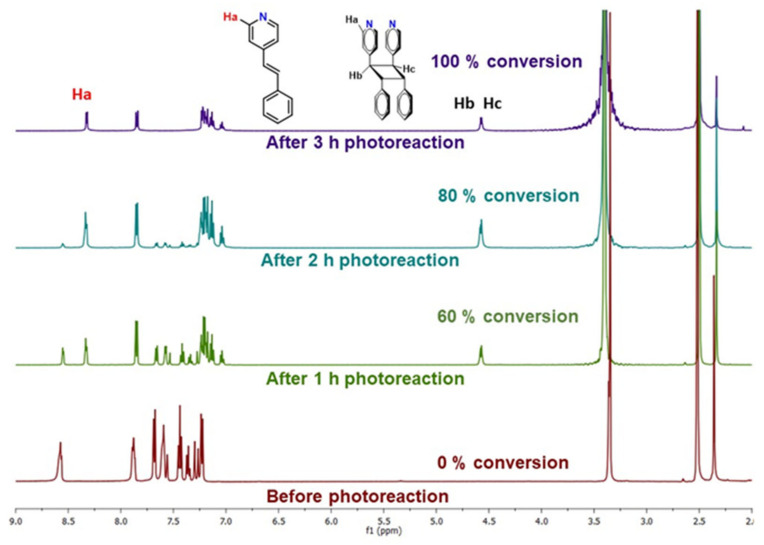
Dimerization of **1** over time followed by ^1^H NMR of **1**.

To confirm the quantitative head-to-head photoreaction, UV-irradiated powdered samples of **1** were carried out in a LUZCHEM photoreactor at different time intervals, and a ^1^H NMR study was performed by dissolving the sample in DMSO-*d_6_*. The formation of the photoproduct was confirmed by the shift of pyridyl protons from 8.56 ppm to 8.33 ppm and the appearance of the characteristic cyclobutane peak near 4.5 ppm, as observed from the time dimerization NMR plot shown in (Figure 3). 

**Figure 4:**
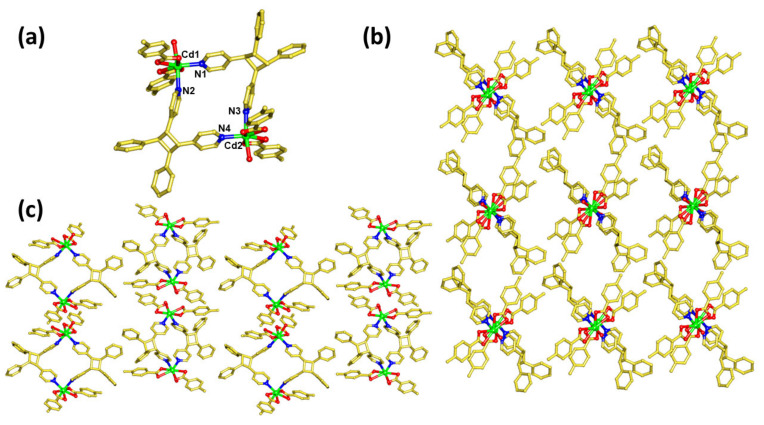
Crystal structure of [Cd_2_(*p*-tol)_4_(*rctt*-ppcb)_2_(H_2_O)_2_] (**4r**). (**a**) Photoproduct of **1** and (**b**,**c**) different perspectives of the arrangement of **1**.

To further confirm the quantitative photoreaction and HH photoreaction, the photoproduct of 1, 4 (photoproduct of 1) is recrystallized from a DMF–methanol solution in a 2:1 ratio. This recrystallized photoproduct has the molecular formula [Cd_2_(*p*-tol)_4_(*rctt*-ppcb)_2_(H_2_O)_2_] (4r, the recrystallized compound of 4). The SCXRD analysis confirmed that the asymmetric unit consists of exactly half of the formula unit, and that the compound is crystallized in the orthorhombic *P*ca2_1_ space group with *Z* = 4. This recrystallized compound is a binuclear complex, where the two Cd(II) atoms are present in pentagonal bipyramidal geometry, and the equatorial sites are coordinated by four O-atoms from *p*-tol in a chelated manner, and *N*-atoms from *rctt*-ppcb (Figure 4a). The axial sites are coordinated by *N*-atoms from *rctt*-ppcb and aqueous molecules, which is significantly different from its precursor, 1. In addition, the long-range arrangement of 4r shows an ABAB type of pattern (Figure 4b). 

### 2.3. Structural Description of [Cd_2_(4FBA)_4_(4spy)_4_] (***2***)

The block-shaped, yellow-colored single crystals of **2** were obtained by following the similar procedure as **1**, using 4FBA instead of *p*-toluate. The compound was crystallized in the triclinic *P*-1 space group with *Z* = 1, and this structure is isostructural to **1** (Figure S6). The comparison of unit cell parameters also shows that both of these compounds are isostructural. This is further confirmed by comparing the molecular arrangement and packing behavior. The alignment of the 4spy molecules is also very similar, with the centroids of the olefin units of 4spy linkers separated by a distance of 3.88 Å and 4.23 Å in the case of HH and HT, respectively. The olefinic hydrogen and the hydrogen of the phenyl group interact with the carboxyl oxygen of 4FBA (C-H···O = 2.34 Å and C-H···O = 2.52 Å, respectively). Also, the pyridyl hydrogen and phenyl hydrogen of 4spy interact with the fluorine atom of 4FBA (C-H···F = 2.56 Å and C-H···F = 2.52 Å, respectively) (Figure S9). The photoreactivity of this compound is also very similar to that of **1**, confirming the intramolecular photoreaction. This confirmation is supported through the quantitative photoreaction analysis of **2** (Figure S4).

### 2.4. Structural Description of [Cd_2_(3FBA)_4_(4spy)_4_] (***3***)

The block-shaped, yellow-colored single crystals of 3 were obtained by following a similar synthetic protocol, by using 3FBA instead of 4FBA. The compound was crystallized in the triclinic *P*-1 space group with *Z* = 1 (Figure S7). However, after a detailed analysis of the compound and its packing, it can be easily observed that this compound also has very similar packing to that of 1 and 2. The alignment of the 4spy linkers is also very similar, whereby the centroids of the olefins that are aligned in HH are separated by 4.06 Å, and those in HT are separated by 3.94 Å. The olefinic hydrogen and the hydrogen of the phenyl group interact with the carboxyl oxygen of 3FBA (C-H···O= 2.42 Å and C-H···O= 2.62 Å, respectively). Also, the olefinic hydrogen of 4spy interacts with the fluorine atom of 3FBA (C-H···F= 2.55 Å). The photoreaction of this compound also has been studied, similar to the previous compounds (Figure S5). This compound also shown quantitative intramolecular photoreaction, confirming the photoreaction within the complex (0D to 0D), and no polymerization has taken place. 

### 2.5. Photoluminescence

**Figure 5:**
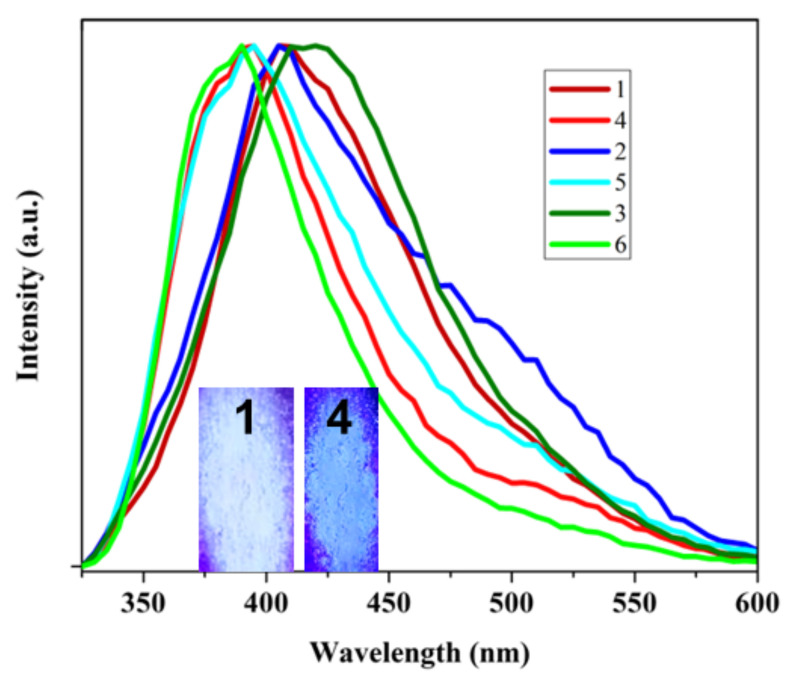
PL spectra of compound **1**, **2** and **3** (before photoreaction) and **4**, **5** and **6** (photo products of **1**, **2** and **3**, respectively) when excited at 310 nm. Inset shows photos of powders **1** and **4**.

Photoluminescence studies have been performed for all these three compounds, both before and after UV-irradiation. Compound **1** showed a fluorescence emission peak at 405 nm when excited using a 310 nm wavelength. After UV-irradiation, this luminescence peak shifted to a lower wavelength at 395 nm, showing a blue shift in the emission peak due to the photoreaction. A similar blue shift was observed for both compounds **2** and **3**. For **2**, the emission peak also shifted from 405 nm to 395 nm (λ_ex_ = 310 nm) after the photoreaction, and, in the case of **3**, the emission peak at λ_max_~420 nm (λ_ex_ = 310 nm) shifted to a lower wavelength at ~390 nm again, showing a blue shift, as shown in Figure 5. This luminescence behavior is mainly due to the charge transfer between the metal-ion and pyridyl part of the 4spy linker. The photoreaction in this 4spy linker leads to the disruption of delocalization, resulting in a change in charge-transfer behavior and causing a blue shift in luminescence after the photoreaction [36,37,38]. 

### 2.6. Thermal Stability

**Figure 6:**
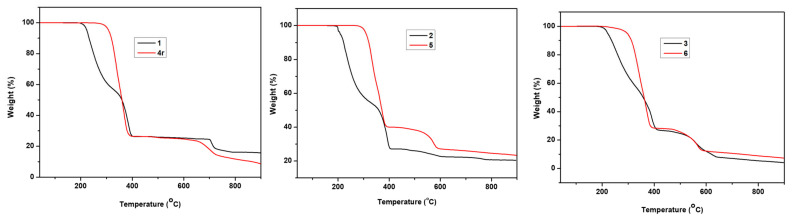
TGA curves of compound **1**, **2** and **3** (before photoreaction) and **4**, **5** and **6** (photo products of **1**, **2** and **3**, respectively).

Meanwhile, TGA studies have been performed for all the three compounds: **1**, **2** and **3**. Compound **1** started to decompose at 200 °C and **2** decomposed at 195 °C, while **3** decomposed at 195 °C, as shown in (Figure 6). After the photoreaction, the decomposition temperatures of the three compounds, labelled as **4r**, **5** and **6**, have increased up to 280 °C, 285 °C and 235 °C, respectively. This further indicates that the enhanced thermal stability of the compound might be due to the formation of a cyclobutane derivative, *rctt*-ppcb. These photoproduct molecules, having larger molecular weight, contribute to enhanced thermal stability when compared to the unreacted or monomer counterparts of the compound.

## 3. Materials and Methods

### 3.1. General 

All the chemicals and solvents were of reagent grade, purchased from different commercial sources and used without any further purification. Powder X-ray diffraction (PXRD) data were recorded on a Bruker Advance X-ray diffractometer with graphite monochromatized Cu Kα radiation (*λ* = 1.54056 Å) at room temperature (298 K). Range: 5–50°, step size: 0.02, number of steps: 2250 and time per step: 0.5 s. ^1^H NMR spectra were recorded on a 400 MHz Bruker DRX 400 spectrometer by calibrating the residual solvent as the reference in DMSO-*d*_6_ solution. The photoluminescence spectral measurements were conducted on a HORIBA Jobin Yvon FluoroMax-4 spectrofluorometer by packing the ground single crystals between the slides, which were placed vertically in the sample holder using λ_ex_ = 310 nm. All UV-irradiation investigations were accomplished using a LUZCHEM UV reactor with the λ_ex_ = 365 nm. Approximately 10 mg of powdered crystalline powder was packed between the glass slides and held vertically inside the photoreactor to ensure uniform exposure to UV radiation from both sides. 

### 3.2. X-ray Crystallography 

Suitable crystals of **1**, **2**, **3** and **4r** were selected and mounted on a Mitegen loop using Paratone-N oil on a SuperNova, Dual, Cu at home/near, AtlasS2 diffractometer. The crystal was kept at 100(1) K during data collection. Using Olex2 [39], the structure was solved with the SHELXT [40] structure solution program, using Intrinsic Phasing, and refined with the SHELXL [41] refinement package, using Least Squares minimization. The CIF files have been deposited with the CCDC and can be accessed free-of-charge (Table 1).

### 3.3. Synthesis of 4spy Linker

**Figure 7:**
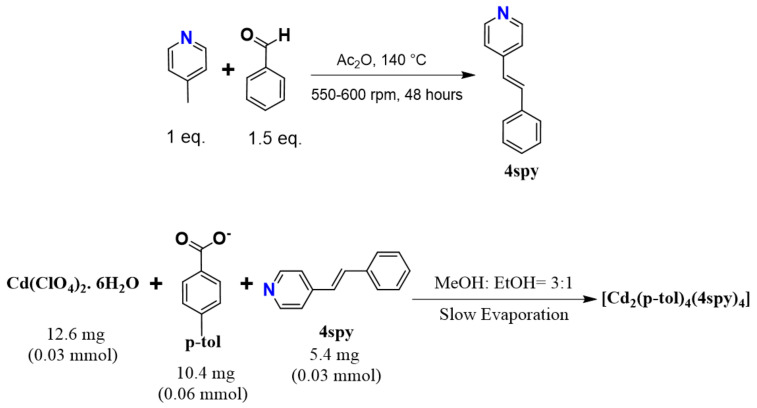
Schematic view showing the synthetic procedure of the 4spy linker (above) and the metal complex, **1** (below).

4spy (4-styrylpyridine) was prepared following a reported procedure (Figure 7) [42,43]. The synthesis involved refluxing a mixture of 4-picoline and benzaldehyde in 1: 1.5 molar ratios with acetic anhydride (20 mL) in a sealed pressure tube at 140 °C and 550–600 rpm for 48 h. At the end of the reflux process, major amounts of acetic acid and acetic anhydride were removed through the addition of an excess amount of water. The mixture was made basic and filtered. The yellow solid compound was collected, dried and purified using column chromatography.

### 3.4. Preparation of Salts

*p*-tol, 4FBA and 3FBA salts were synthesized in bulk amounts by combining equimolar ratios of potassium hydroxide with the respective acid solutions. The solution mixture was adjusted to a medium basic pH and kept at 110 °C in a hot air oven for 72 h. The salt was collected after the complete evaporation of the solvent.

### 3.5. Preparation of [Cd_2_(p-tol)_4_(4spy)_4_] (***1***)

Block-shaped yellow single crystals of **1** were obtained through the slow evaporation method, where Cd(ClO_4_)_2_·6H_2_O (12.6 mg, 0.03 mmol), potassium salt of *p*-toluate (10.4 mg, 0.06 mmol) and 4spy (5.4 mg, 0.03 mmol) were mixed in 2 mL of 3:1 MeOH/EtOH solution. The crystals were collected before all the solvent evaporated to avoid impurities. ^1^H NMR analysis was conducted (DMSO-*d*_6_, 400 MHz, 298 K): δ 8.57 ppm (d, 4H, pyridyl protons of 4spy), 7.22–7.89 ppm (m, 7H, aromatic protons of 4spy). Elemental analysis was conducted: C, 67.70; H, 4.87; N, 3.76 (calculated); C, 60.70; H, 4.54; N, 3.38 (measured). 

### 3.6. Preparation of [Cd_2_(4FBA)_4_(4spy)_4_] (***2***)

Block crystals were obtained through the slow evaporation method, containing Cd(ClO_4_)_2_ (12.6 mg, 0.03 mmol), potassium salt of 4FBA (10.5 mg, 0.06 mmol) and 4spy (5.4 mg, 0.03 mmol) in 2 mL of MeOH/EtOH solution. The crystals were collected before all the solvent evaporated to avoid impurities. ^1^H NMR analysis was conducted (DMSO-*d*_6_, 400 MHz, 298 K): δ 8.55 ppm (d, 4H, pyridyl protons of 4spy), 7.19–7.66 ppm (m, 7H, aromatic protons of 4spy). Elemental analysis was conducted: C, 63.80; H, 4.02; N, 3.72 (calculated); C, 62.40; H, 3.88; N, 3.34 (measured).

### 3.7. Preparation of [Cd_2_(3FBA)_4_(4spy)_4_] (***3***)

Block crystals were obtained through the slow evaporation method containing Cd(ClO_4_)_2_ (12.6 mg, 0.03 mmol), potassium salt of 3FBA (10.44 mg, 0.06 mmol) and 4spy (5.4 mg, 0.03 mmol) in 2 mL of MeOH/EtOH solution. ^1^H NMR analysis was conducted (DMSO-*d*_6_, 400 MHz, 298 K): δ 8.55 ppm (d, 4H, pyridyl protons of 4spy), 7.19–7.66 ppm (m, 7H, aromatic protons of 4spy). Elemental analysis was conducted: C, 63.80; H, 4.02; N, 3.72 (calculated); C, 63.14; H, 3.89; N, 3.56 (measured).

### 3.8. Preparation of [Cd_2_(p-tol)_4_(rctt-ppcb)_2_(H_2_O)_2_] (4r, Recrystallized Compound of 4 (Photoproduct of ***1***))

Powdered **1** (5 mg), placed between two glass slides, was irradiated inside a UV reactor of wavelength 365 nm for 48 h. Further, the irradiated sample was dissolved in a 2:1 ratio of DMF-methanol solution. The crystals were collected before all the solvent evaporated to avoid impurities. ^1^H NMR analysis was conducted (DMSO-*d*_6_, 400 MHz, 298 K): δ 8.55 ppm (d, 4H, pyridyl protons of 4spy), 7.19–7.66 ppm (m, 7H, aromatic protons of 4spy).

## 4. Conclusions

To sum up, we have successfully synthesized three novel binuclear Cd(II) isostructural complexes using three benzoate derivatives (two para-derivatives, and one meta-derivative) as co-linkers, with 4spy serving as the photoreactive ligand. All these three complexes are isostructural in nature, confirmed through the careful analyses of unit cell parameters, as well as the packing arrangement and alignment of 4spy systems. In all these three complexes, the 4spy system is aligned both in HH and in HT manners, and the olefins of these 4spy linkers follow Schmidt’s criteria. The percentage of photoreaction has been used as a tool to confirm the photo-transformation in an HH manner (0D to 0D), unlike the photo-polymerization of metal complexes. In addition, this HH photoreaction also has been confirmed through the recrystallization of the photoproduct. This [2+2] photoreaction led to an increment in the thermal stability of the compound, confirmed through TGA analysis. The fluorescence studies of these compounds showed the blue shift of the luminescence peaks during the photoreaction.

## Figures and Tables

**Table 1:** Crystal and experimental data and refinement parameters of **1**, **2**, **3** and **4r**.

	1	2	3	4r
CCDC	2294502	2294503	2294504	2294505
Empirical formula	C_84_H_72_Cd_2_N_4_O_8_	C_80_H_60_Cd_2_F_4_N_4_O_8_	C_80_H_60_Cd_2_F_4_N_4_O_8_	C_84_H_76_Cd_2_N_4_O_10_
Formula weight	1490.25	1506.12	1506.12	1526.28
Temperature/K	99.97(10)	99.96(11)	292.32(13)	99.9(6)
Crystal system	triclinic	triclinic	triclinic	orthorhombic
Space group	*P*-1	*P*-1	*P*-1	*P*ca2_1_
a/Å	10.5703(2)	10.64970(10)	11.00600(10)	14.7172(2)
b/Å	11.2955(2)	10.86120(10)	11.18380(10)	14.2024(2)
c/Å	15.7407(3)	15.7523(2)	16.00910(10)	33.0645(5)
α/°	106.856(2)	106.3610(10)	94.0310(10)	90
β/°	92.083(2)	92.6840(10)	106.6310(10)	90
γ/°	109.025(2)	110.9190(10)	112.9150(10)	90
Volume/Å^3^	1682.51(6)	1610.79(3)	1701.83(3)	6911.13(17)
Z	1	1	1	4
ρ_calc_ g/cm^3^	1.471	1.553	1.470	1.467
μ/mm^−1^	5.570	5.919	5.603	5.460
Goodness-of-fit on F^2^	1.047	1.063	1.065	1.051
Final R indexes [*I* > 2*σ*(*I*)]	*R*_1_ = 0.0349, *wR*_2_ = 0.0923	*R*_1_ = 0.0328, *wR*_2_ = 0.0877	*R*_1_ = 0.0409, *wR*_2_ = 0.1090	*R*_1_ = 0.0674, *wR*_2_ = 0.1851

## Data Availability

The data presented in this study are available in the Supplementary Materials.

## Supplementary Materials

The following supporting information can be downloaded at: https://www.mdpi.com/article/10.3390/molecules29020351/s1, Figure S1. Comparison of SCXRD simulated & experimental PXRD pattern of 1.; Figure S2. Comparison of SCXRD simulated & experimental PXRD pattern of 2.; Figure S3. Comparison of SCXRD simulated & experimental PXRD pattern of 3.; Figure S4. Time-dependent 1H NMR plot of 2.; Figure S5. Time-dependent 1H NMR plot of 3.; Figure S6. Crystal structure of [Cd2(4FBA)4(4spy)4] (2). (a) Metal complex of 2. (b) Alignment of the photoreactive linker in 2. (c) Extended head-to-head and head-to-tail alignment. All H-atoms are omitted for clarity. Colour codes: C = golden; N = dark blue; O = red; F= cyan; Cd = green.; Figure S7. Crystal structure of [Cd2(3FBA)4(4spy)4] (3). (a) Metal complex of 3. (b) Alignment of the photoreactive linker in 3. (c) Extended head-to-head and head-to-tail alignment. All H-atoms are omitted for clarity. Colour codes: C = golden; N = dark blue; O = red; F= cyan; Cd = green.; Figure S8. Depiction of non- covalent interactions in [Cd2(p-tol)4(4spy)4] (1). (a) π-π interactions are shown as blue shade. C-H---π interactions are shown as green dashed line. C-H---O interactions are shown as blue.(dark and light) and red dashed line. (b) Enlarged view of photoreactive linker depicting non- covalent interactions. All H-atoms are omitted for clarity. Colour codes: C = golden; N = dark blue; O = red; Cd = green.; Figure S9. Depiction of non- covalent interactions in [Cd2(3FBA)4(4spy)4] (3). (a) C-H---F interactions are shown as blue dashed line. C-H---O interactions are shown as black and green dashed line. (b) Enlarged view of photoreactive linker depicting non- covalent interactions. All H-atoms are omitted for clarity. Colour codes: C = golden; N = dark blue; O = red; F= cyan; Cd = green. Table S1. Bond lengths along the coordination geometry of Cd(II) ion.
