# Supplementary material for: Solid-State [2+2] Photoreaction of Isostructural Cd(II) Metal Complexes and Solid-State Fluorescence
Source: Molecules. 2024 Jan 10;29(2):351. doi: 10.3390/molecules29020351 (PMC10820883; doi:10.3390/molecules29020351)
Supplement: Supplementary file 1 [file molecules-29-00351-s001.zip › molecules-2733738-supplementary-done.pdf]

Supplementary Material

Solid-State [2+2] Photoreaction of Isostructural Cd(II) Metal Complexes and Solid-State Fluorescence

Akansha Ekka [1], Aditya Choudhury [1], Madhumita Samanta [1], Ayushi Deshmukh [1], Nathan R. Halcovitch [2], In-Hyeok Park [3,*] and Raghavender Medishetty [1,*]

[1] Department of Chemistry, Indian Institute of Technology Bhilai, Kutelabhata, Durg 491001, India
[2] Chemistry Department, Lancaster University, LA1 4YB, United Kingdom
[3] Graduate School of Analytical Science and Technology (GRAST), Chungnam National University, Daejeon 34134, Republic of Korea
* Correspondence: ipark@cnu.ac.kr (I.-H.P.); raghavender@iitbhilai.ac.in (R.M.)

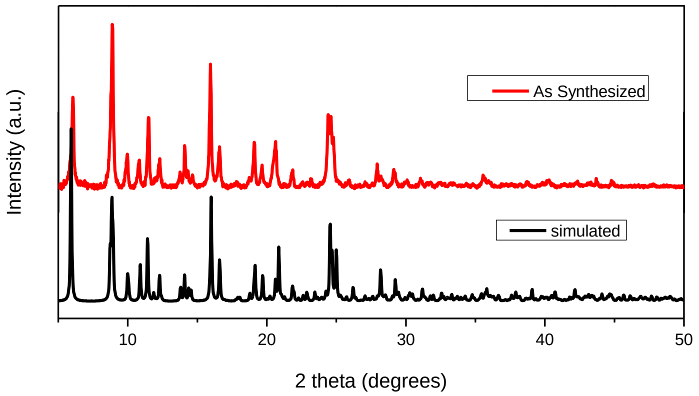

Figure S1. Comparison of SCXRD simulated & experimental PXRD pattern of **1.**

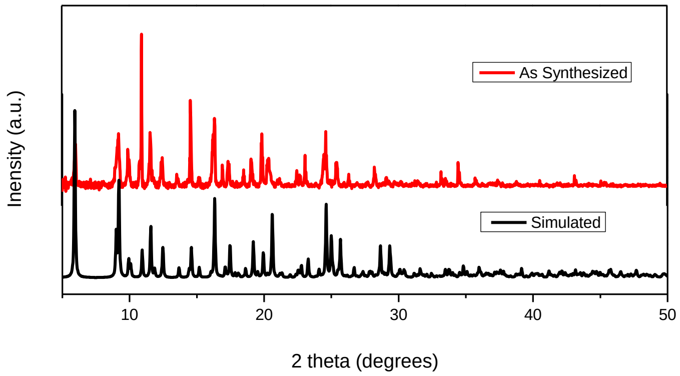

Figure S2. Comparison of SCXRD simulated & experimental PXRD pattern of **2.**

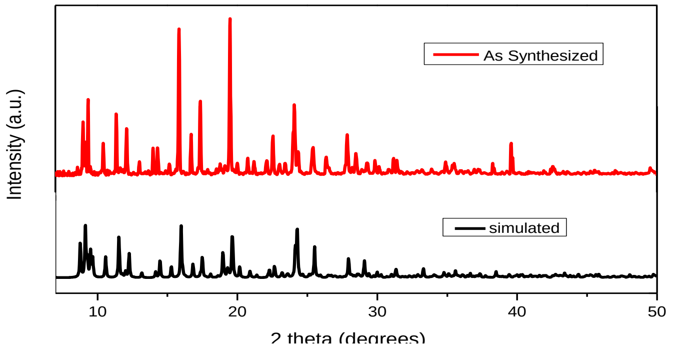

Figure S3. Comparison of SCXRD simulated & experimental PXRD pattern of **3.**

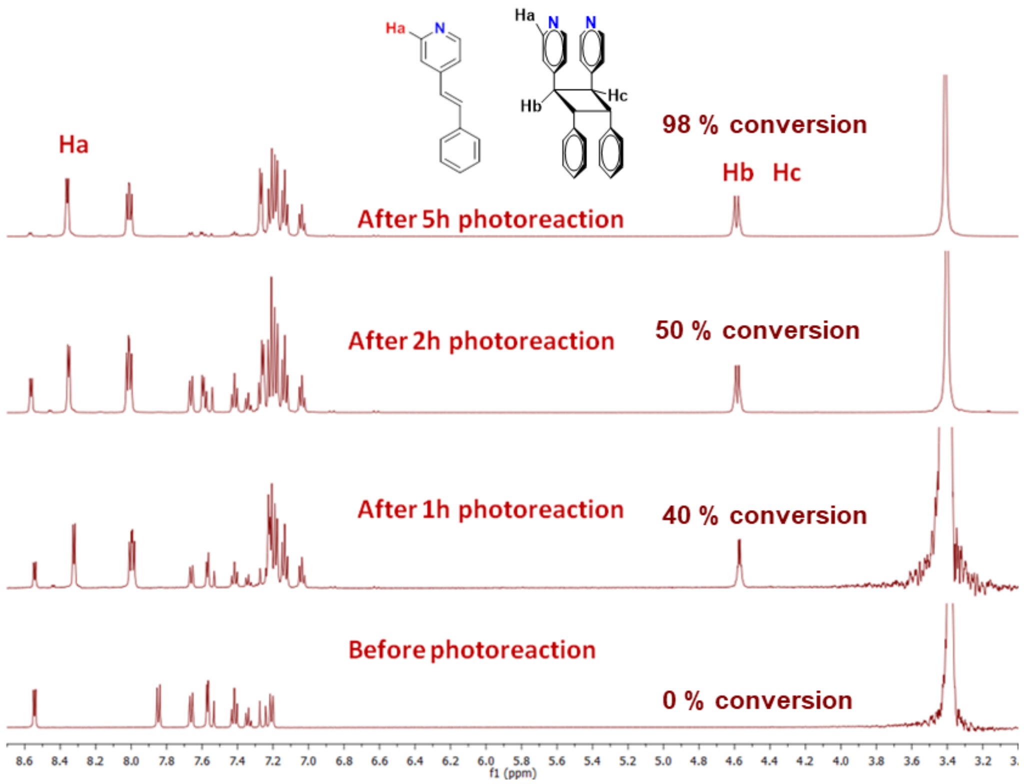

Figure S4. Time-dependent ^{1}H NMR plot of **2**.

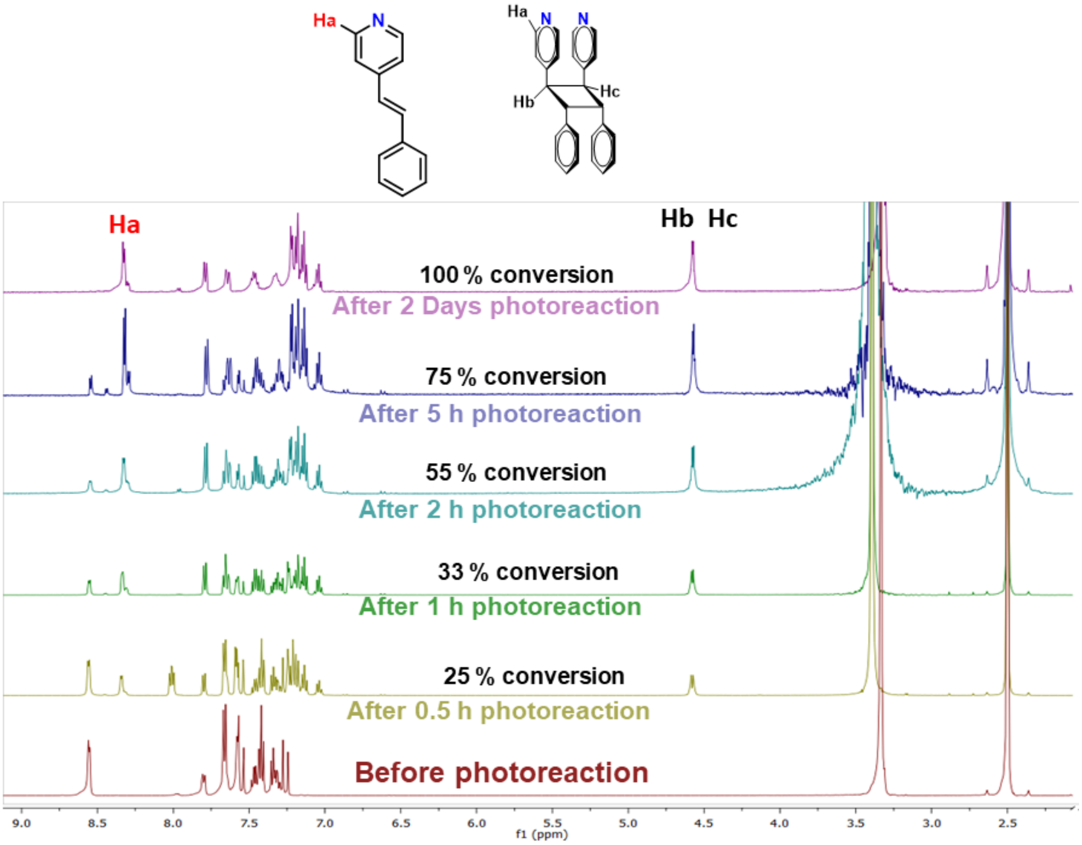

Figure S5. Time-dependent ^{1}H NMR plot of **3**.

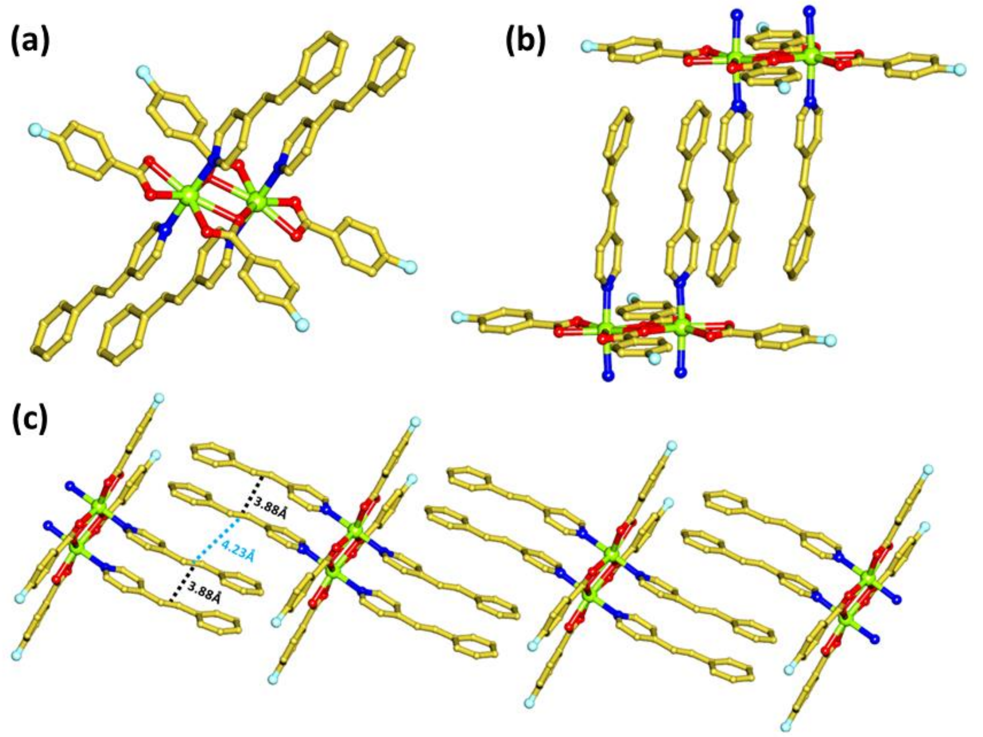

Figure S6. Crystal structure of [$Cd_2(4FBA)_4(4spy)_4$] (**2**). **(a)** Metal complex of **2**. **(b)** Alignment of the photoreactive linker in **2**. **(c)** Extended head-to-head and head-to-tail alignment**.** All H-atoms are omitted for clarity. Colour codes: C = golden; N = dark blue; O = red; F= cyan; Cd = green.

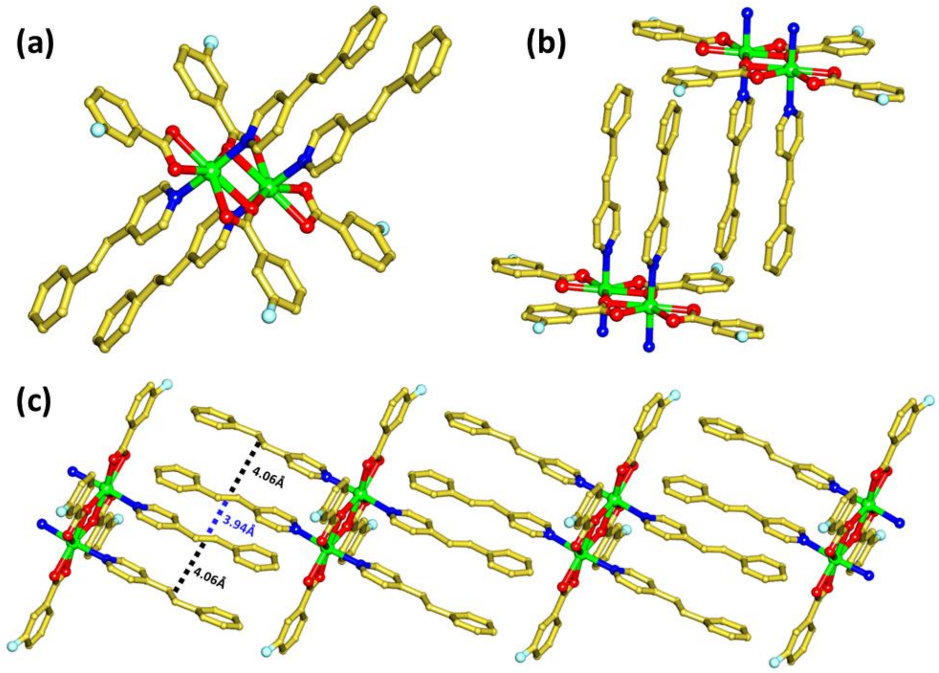

Figure S7. Crystal structure of [$Cd_2(3FBA)_4(4spy)_4$] (**3**). **(a)** Metal complex of **3**. **(b)** Alignment of the photoreactive linker in **3**. **(c)** Extended head-to-head and head-to-tail alignment**.** All H-atoms are omitted for clarity. Colour codes: C = golden; N = dark blue; O = red; F= cyan; Cd = green.

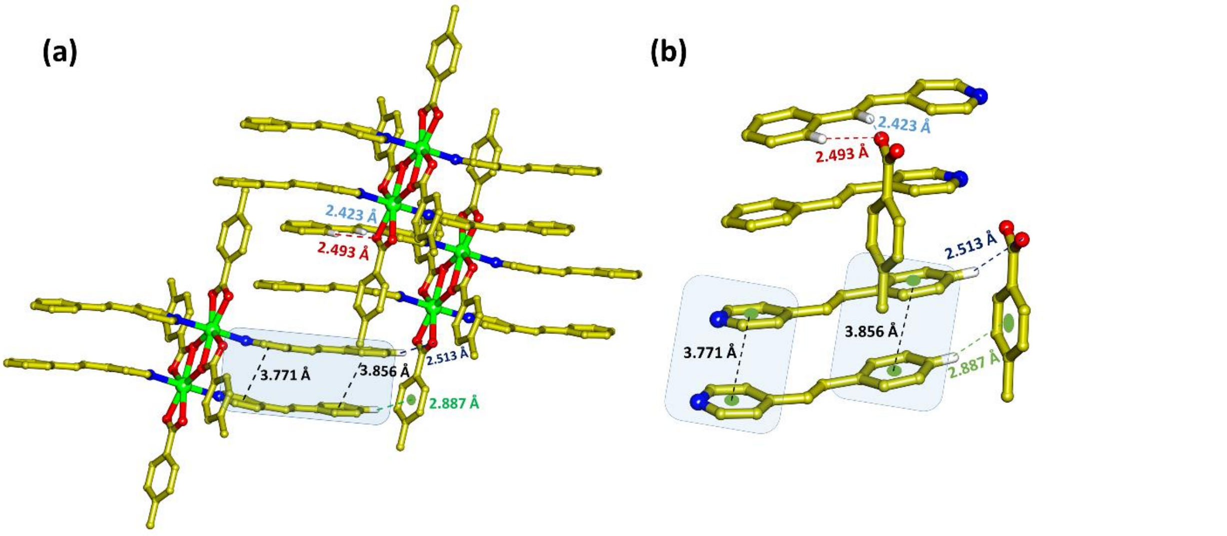

Figure S8. Depiction of non- covalent interactions in [Cd2(p-tol)4(4spy)4] (**1**). (a) π-π interactions are shown as blue shade. C-H---π interactions are shown as green dashed line. C-H---O interactions are shown as blue .(dark and light) and red dashed line. (b) Enlarged view of photoreactive linker depicting non- covalent interactions. All H-atoms are omitted for clarity. Colour codes: C = golden; N = dark blue; O = red; Cd = green.

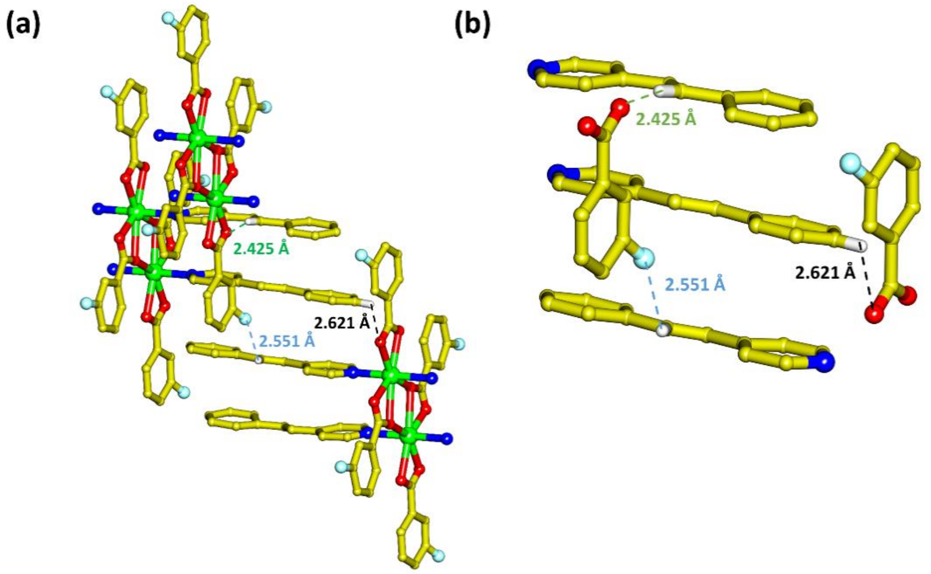

Figure S9. Depiction of non- covalent interactions in [Cd2(3FBA)4(4spy)4] (3). (a) C-H---F interactions are shown as blue dashed line. C-H---O interactions are shown as black and green dashed line. (b) Enlarged view of photoreactive linker depicting non- *covalent interactions.* All H-atoms are omitted for clarity. Colour codes: C = golden; N = dark blue; O = red; F= cyan; Cd = green.

Table S1. Bond lengths along the coordination geometry of Cd(II) ion.

Compound-1:

Bond between	Length (Å)
Cd1-O1	2.4234(17)
Cd1-O2	2.3566(16)
Cd1-O3	2.3358(17)
Cd1-O4	2.5125(17)
Cd1-O4	2.3614(16)
Cd1-N1	2.325(2)
Cd1-N2	2.316(2)

Compound-2:

Bond between	Length (Å)
Cd1-O1	2.3181(15)
Cd1-O2	2.5894(15)
Cd1-O2	2.3317(14)
Cd1-O3	2.3832(14)
Cd1-O4	2.4033(15)
Cd1-N1	2.3072(18)
Cd1-N2	2.296(6)

Compound-3:

Bond between	Length (Å)
Cd1-O1	2.352(3)
Cd1-O2	2.378(2)
Cd1-O2	2.529(3)
Cd1-O3	2.404(3)
Cd1-O4	2.379(3)
Cd1-N1	2.320(3)
Cd1-N2	2.327(3)

Compound-4r:

Bond between	Length (Å)
Cd1-O1	2.426(7)
Cd1-O2	2.389(9)
Cd1-O3	2.373(9)
Cd1-O4	2.469(12)
Cd1-O5	2.352(7)
Cd1-N1	2.329(8)
Cd1-N2	2.355(8)
Cd2-O6	2.311(10)
Cd2-O7	2.631(14)
Cd2-O8	2.407(8)
Cd2-O9	2.398(10)
Cd2-O10	2.340(7)
Cd2-N3	2.324(9)
Cd2-N4	2.326(8)
